# Microcatheter–Assisted Circumferential Trabeculotomy in Primary Congenital Glaucoma: Long-Term Clinical Outcomes

**DOI:** 10.3390/jcm11020414

**Published:** 2022-01-14

**Authors:** Luigi Fontana, Alberto Neri

**Affiliations:** 1 Ophthalmology Unit, DIMES, Alma Mater Studiorum, Ophthalmology Department, University of Bologna and S. Orsola-Malpighi Teaching Hospital, 40138 Bologna, Italy; 2 Ophthalmology Unit, Azienda USL—IRCCS di Reggio Emilia, 42122 Reggio Emilia, Italy; neri.mail2@gmail.com

**Keywords:** primary congenital glaucoma, circumferential trabeculotomy, microcatheter, long-term outcomes

## Abstract

Purpose: The purpose of this study was to report the long-term efficacy and clinical outcomes of microcatheter-assisted circumferential trabeculotomy (MCT) in children with primary congenital glaucoma (PCG). Methods: This is a single-center retrospective study including consecutive children with PCG who underwent MCT with > two years follow up. The primary outcome was surgical success, defined as intraocular pressure (IOP) ≤ 21 mmHg with (qualified) or without (complete) medications, measured at six months, one year, and then annually. Secondary outcomes were visual acuity (VA), refraction, axial length (AXL), complications, reinterventions, and number of medications. Results: Twelve eyes of ten patients were included. In eight children only one eye was affected. The mean ± standard deviation (SD) age at surgery was 6.3 ± 4.1 months. The mean postoperative follow-up was 66 ± 35 months. The mean IOP was 34.3 ± 9.6 mmHg preoperatively and 14.6 ± 2.3 mmHg postoperatively at the last visit (*p* < 0.001). Complete success was achieved at all time points in 10 out of 12 eyes, while 2 eyes had a qualified success. At three years of age, the mean VA of the operated eyes was 0.25 ± 0.12 logMAR, the mean spherical equivalent was −0.78 ± 1.43 diopters, and the mean AXL was 23.78 mm. Transient hyphema was the only complication observed. None of the children required additional glaucoma surgery. Conclusions: Circumferential trabeculotomy for PCG effectively lowers the IOP at more than two years after surgery. Following this procedure, the prognosis for the visual function is good, and the refractive error is low. Postoperative complications were not significant.

## 1. Introduction

Primary congenital glaucoma (PCG) is the most common non-syndromic glaucoma in childhood, occurring in one of 10,000–20,000 live births in western countries [1,2]. In PCG, the whole eye is modified and damaged by high intraocular pressure (IOP) due to isolated trabeculodysgenesis [3]. Angle surgery is the mainstay of treatment of PCG [4,5,6], with the choice between goniotomy and trabeculotomy dictated by corneal clarity, surgeon’s experience, and preference [3,7]. The results of angle surgery are good, with most studies citing a 70% to 90% rate of success for both goniotomy and trabeculotomy [8]. However, approximately 1/3 of the iridocorneal angle is opened with both techniques, often requiring repeated procedures to obtain effective IOP control, using a step-wise approach [8]. Moreover, a metallic trabeculotome probe can create a false passage, causing tissue disruption [9].

In the attempt to improve the efficacy of angle surgery, Beck and Lynch described 360° trabeculotomy using a 6-0 prolene suture [9], modifying a technique introduced by Smith in 1960 [10]. Circumferential trabeculotomy (CTT) showed at least the same success rate of repeated goniotomy or trabeculotomy with the advantage of a single surgical procedure [11]. Lately, the availability of illuminated microcatheters has improved the safety of CTT, avoiding the potentially serious complications of suture misdirection into the suprachoroidal and subretinal space [12]. As a result, microcatheter-assisted circumferential trabeculotomy (MCT) is increasingly used worldwide as primary surgical technique in PCG. Still, the reported outcomes of this procedure are mostly limited to a short-term (12–24 months) [12,13,14,15]. Herein we report the long-term clinical outcomes of MCT for PCG in a tertiary referral center.

## 2. Materials and Methods

### 2.1. Settings

In this single-center retrospective study, we analyzed the clinical outcomes of patients with PCG treated by MCT at the Ophthalmology Unit of Azienda USL–IRCCS di Reggio Emilia (Reggio Emilia, Italy) from January 2011 to January 2021. The study was approved by the local Institutional Review Board (registration number: 170/2021/OSS/AUSLRE) for the collection of clinical data and was conducted in accordance with the principles of the Declaration of Helsinki. In addition, written informed consent was obtained from the parents of eligible children after a thorough explanation of the risk and benefits of the procedure.

### 2.2. Participants

Clinical data were extracted from the hospital records. Data from children with at least two years of follow-up after surgery were included for the analysis. Patients with shorter follow-up, other types of pediatric glaucoma, previous ocular surgeries, and coexisting ocular pathologies were excluded.

### 2.3. Data Collection

The data collected included basic demographic information, complete eye examination at baseline and at each follow-up visit, glaucoma medications, surgical procedure details, operative complications, late complications, and reinterventions. Eye examination included IOP values and measurement technique, slit lamp examination, gonioscopy and fundoscopy, refraction, visual acuity (VA) (Logarithm of the Minimum Angle of Resolution, LogMAR), and stereopsis (Lang Stereotest). In unilateral cases, clinical data of the fellow eyes were also collected.

The primary outcome was IOP measured at 6 months, 12 months, and then annually. Success was defined as postoperative IOP > 5 and ≤21 mmHg, with an IOP reduction > 20% from baseline, measured at each postoperative time point, with (qualified success) or without (complete success) glaucoma drops. Eyes were classified as failing if IOP > 21 was measured on two consecutive study visits despite maximum glaucoma therapy or if additional glaucoma surgery was performed. Secondary outcomes were complications, reinterventions, number of medications after surgery, axial length, VA (LogMAR), and refraction.

Baseline and postoperative clinical measurements were conducted by two observers (AN, LF). During examination under anesthesia (EUA), IOP was measured in the early phase of anesthesia (sevoflurane) induction before intubation using a hand-held Perkins applanation tonometer (Perkins Tonomter MK2; Clement Clark Ophthalmic, Harlow, UK) or a hand-held rebound tonometer (ICare Pro, Icare Finland OY, Helsinki, Finland) in case of corneal edema precluding applanation tonometry. In addition, a-scan axial length measurements were conducted at each EUA visit (Compact Touch, Quentel Medical, France).

### 2.4. Surgical Technique

**Figure 1:**
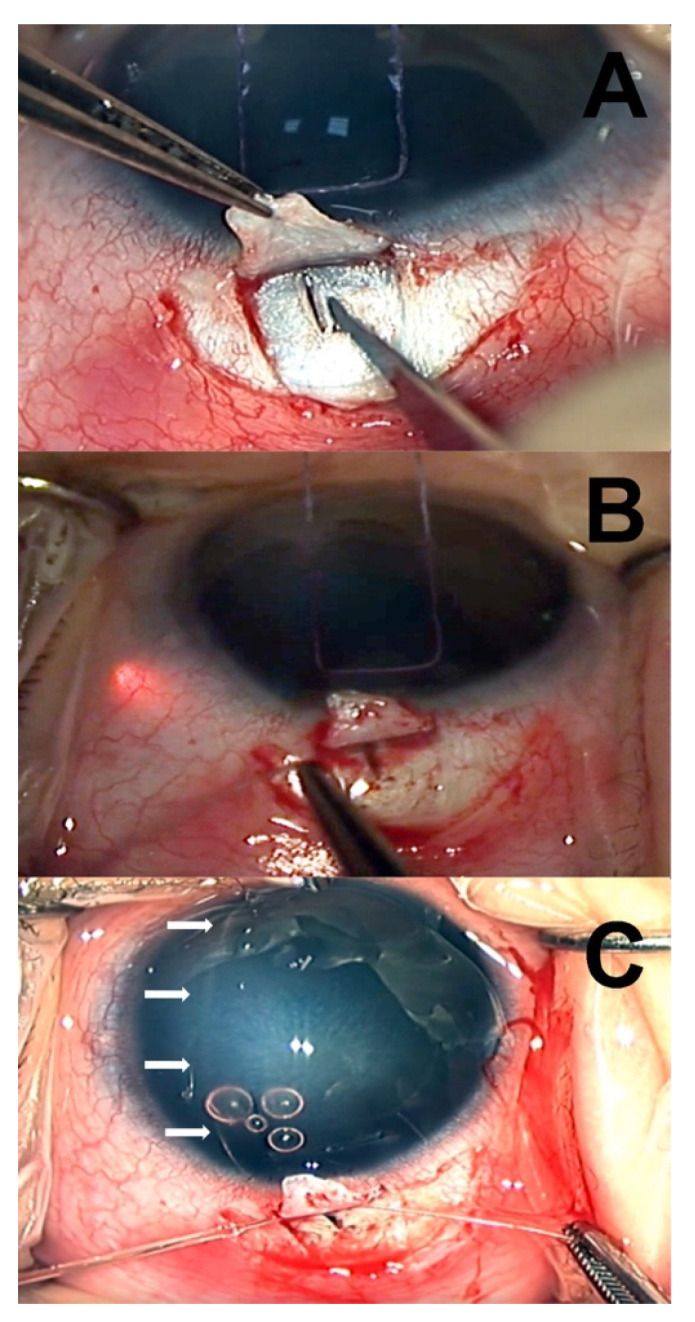
Surgical technique. (**A**) Following conjunctival peritomy, a limbus-based scleral flap is fashioned, then a radial cut in the deep sclera is performed with a slit knife, posteriorly to the limbus, and progressively deepened until the outer wall of the Schlemm canal (SC) is cut. (**B**) An illuminated microcatheter (iTrack 250A; iScience Interventional, Menlo Park, CA, USA) is advanced along the canal circumferentially for 360°. (**C**) Once circumnavigation of the SC is accomplished, both ends of the microcatheter are pulled, creating a 360° internal opening of the SC and trabecular meshwork.

All of the surgical procedures were performed by a single clinician (LF) under general anesthesia. The surgical technique used was similar to the one described by Sarkisian et al. [12], using a temporal approach (Figure 1). First, a limbal stay suture was applied using a spatulated 6-0 vicryl. After a temporal conjunctival peritomy, a limbus-based superficial scleral flap of 4 × 4 mm was fashioned using a crescent blade. Then, a radial cut in the deep sclera was performed with a slit knife, posteriorly to the limbus, and progressively deepened until aqueous percolation was observed. Schlemm’s canal (SC) was identified by introducing a 5.0 prolene suture cauterized at the tip. Prolene suture was then removed, and an illuminated microcatheter (iTrack 250A; iScience Interventional, Menlo Park, CA) was threaded into the SC and gently advanced along the canal circumferentially for 360°.

In cases where the microcatheter tip met with an obstruction within SC or became misdirected, catheterization was attempted in the opposite direction, and viscoelastic was injected through the microcatheter in attempt to dilate the canal. If SC could not be identified, catheterization was tried at a different quadrant (supero-temporal or infero-temporal). Once circumnavigation of the SC was accomplished, the anterior chamber was deepened by injecting dispersive viscoelastic (Eyefill, Bausch and Lomb, Bridgewater, NJ, USA), and both ends of the microcatheter were pulled, creating a 360° internal opening of the SC and TM. Finally, the catheter was removed, and the scleral flap was secured using a 10-0 vicryl suture to prevent the creation of a filtering bleb.

After surgery, topical therapy included pilocarpine 10 mg/dL eye drops TID for three weeks, dexamethasone 0.1% and tobramycin 0.3% eye drops combined, administered every three hours during the first week and then tapered progressively during the following three weeks according to the surgeon’s discretion. Patients were seen on days 1, 7 and 14 after surgery to observe the anterior chamber for shallowing and hyphema. EUAs were performed at one month, three months, six months, and yearly thereafter.

### 2.5. Statistical Analysis

Statistical analysis was performed with Microsoft Excel v. 16.42 (Microsoft Corporation, Redmond, WA, USA) and Statplus:mac v. 8 (AnalystSoft, Walnut, CA, USA). Kolmogorov-Smirnov Test (with Lilliefors correction) was used to assess if data had a normal distribution. Then, for data with normal distribution, paired t-test was used to compare repeated measurements of the same eyes, and independent t-test was used to compare affected and unaffected eyes. The Mann-Whitney test was used for data without normal distribution. Kaplan-Meyer survival analysis was performed to analyze the surgical success. Finally, power analysis of results was performed post-hoc using confidence intervals for binomi.

## 3. Results

The records of 14 children with PCG who underwent MCT during the study period were retrieved. Two patients with bilateral glaucoma and cataracts and two patients with follow-up <2 years were excluded. Twelve eyes of ten patients met the inclusion/exclusion criteria mentioned above. In eight children only one eye was affected. The mean age at surgery was 6.2 ± 4.2 months (3.5 ± 0.83 months and 9.0 ± 4.3 months for male and female, respectively), and mean follow-up was 66 ± 35 months (range 24–121 months). All of the children underwent surgery within two weeks of diagnosis.

Characteristics of the patients included into the study are resumed in Table 1. At the time of surgery, mean IOP, axial length, and corneal diameter were 34.33 ± 9.63 mmHg, 22.67 ± 0.67 mm and 13.25 ± 0.39 mm in the affected eyes, and 13.25 ± 4.92 mmHg, 19.62 ± 1.01 mm and 11.75 ± 0.46 mm, in the not affected eyes (*p* < 0.001 for all comparisons). All of the eyes with PCG had corneal edema at the time of surgery, and 8 out of 12 eyes presented Haab striae. Four eyes received treatment with glaucoma drops before surgery (pilocarpine 1% combined with dorzolamide or betaxolol). Glaucoma medications were suspended after surgery in all patients.

A complete 360° trabeculotomy was achieved in all eyes. In three cases, a second scleral incision in the infero-temporal quadrant was required to locate the SC, and in two cases, catheter reinsertion and viscoelastic injection were required to complete canalization. Immediately after surgery, a moderate hyphema was observed in all patients that spontaneously resolved within two weeks. In three cases, transient shallowing of the anterior chamber was noted one week after surgery, while other postoperative complications were not encountered.

**Figure 2:**
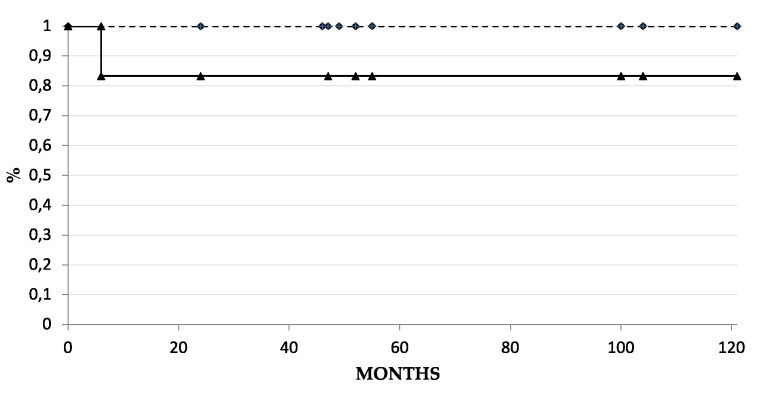
Kaplan-Meier survival analysis for surgical success. Solid line represents complete success (IOP ≤ 21 mmHg without glaucoma medications), dashed line represents qualified success (IOP ≤ 21 mmHg with or without glaucoma medications). Diamonds and triangles represent single patients at their last follow up visit.

In 10 out of 12 eyes, complete success was achieved during the study period, while two eyes had a qualified success, one (case 4) requiring two glaucoma medications (brinzolamide and travoprost), and one (case 9) a single drug (tafluprost). No cases of failure occurred during the time encompassed by this study (alpha = 0.05, CI 93.98–100%). Kaplan–Meier curves for complete and qualified success are shown in Figure 2.

**Figure 3:**
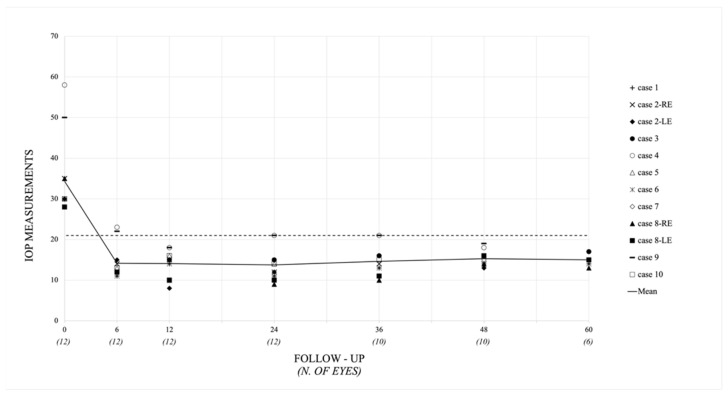
Scatterplot of preoperative and postoperative intraocular pressure (IOP) (mmHg) (*Y* axis) and follow-up (months) (*X* axis). The value of IOP for each patient is represented at different postoperative time intervals. The dashed line indicates the 21 mmHg IOP value. The solid line indicates the mean IOP value. For children who underwent surgery in both eyes, IOP values are represented independently (RE, right eye; LE, left eye). N = number.

**Figure 4:**
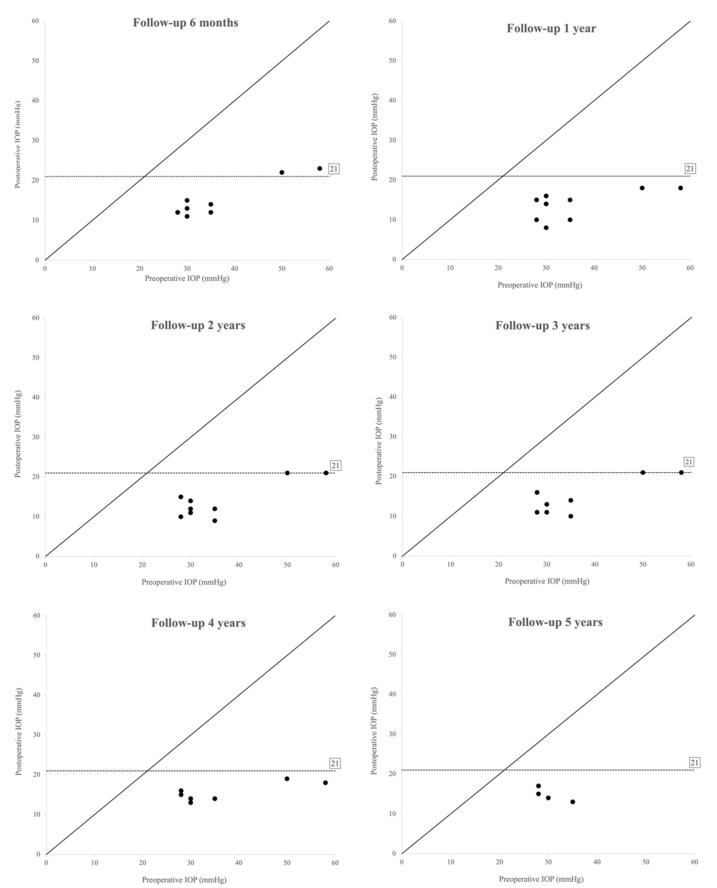
Scatterplot of preoperative (*X* axis) and postoperative (*Y* axis) intraocular pressure (IOP) (mmHg) at six months, one, two, three, four, and five years after surgery. The value of IOP for each operated eye is represented with a black dot. The dashed line indicates the 21 mmHg value. Equality between preoperative and postoperative measurements is represented with a solid line.

Mean postoperative IOP was 14.2 ± 4.1 mmHg at six months, 14.8 ± 3.2 mmHg at one year, 13.7 ± 3.9 mmHg at two years, 14.64 ± 3.7 mmHg at three years, 15.3 ± 1.9 mmHg at four years, and 15.0 ± 2.4 mmHg at five years, while mean IOP of the fellow eyes was 13.5 ± 4.4 mmHg, 15.2 ± 3.0 mmHg, 14.0 ± 4.2 mmHg, 15.7 ± 2.6 mmHg, 15.0 ± 2.4 mmHg, and 14.0 ± 0.0 mmHg, at the same time points (*p* > 0.5 for all comparisons). Figure 3 and Figure 4 show the values of preoperative and postoperative IOP through 5 years of follow-up. At the last evaluation (12 eyes, mean follow-up time 66 ± 35 months), mean postoperative IOP was 14.6 ± 2.2 mmHg, corresponding to a mean IOP lowering of 57.4% from baseline (*p* < 0.001, statistical power 100%). In the non-operated fellow eyes, the mean IOP was 14.2 ± 3.1 mmHg, unvaried from preoperative (*p* > 0.5).

In patients younger than three years, the examiners judged VA measurements unreliable due to poor child cooperation and were not included in the analysis. At three years of age, the mean VA of the operated eyes was 0.25 ± 0.12 logMAR (10 eyes). In unilateral patients (six eyes), mean VA was 0.26 ± 0.12 logMAR and 0.00 ± 0.00 logMAR, in the operated and fellow eyes, respectively (*p* < 0.001). Amblyopia, when present (seven eyes), was treated with spectacles prescription and patching.

Refraction at three years showed a mean spherical equivalent (SE) of −1.06 ± 1.59 and +0.62 ± 1.48 diopters (D), and mean astigmatism of 1.37 ± 1.43 and 0.50 ± 0.65 D, in the affected and fellow eyes, respectively (*p* < 0.05 for all comparisons). At last evaluation (12 eyes, mean follow-up time 66 ± 35 months), the mean SE and refractive cylinder were −0.96 ± 1.93 D and 0.75 ± 1.43 D in the operated eyes, and +1.8 ± 1.01 D and 1.06 ± 0.77 D in fellow eyes. At the same time point, the mean axial length was 23.78 ± 0.32 mm and 21.91 ± 0.10 mm in the affected and fellow eyes, respectively (*p* < 0.001).

Strabismus was found in 5 out of 10 patients, four with unilateral and one with bilateral PCG. Among the patients with strabismus, two had esotropia, one exotropia, and two exophoria/tropia. One patient with esotropia had strabismus surgery at six years. Stereopsis was present in 7 out of 10 patients.

## 4. Discussion

PCG is a blinding condition with challenging treatment, where the first operation chosen is often the best chance of long-term success for the patient [4,5,6,7].

The current study found that MCT successfully lowered the IOP in PCG patients at more than two years after surgery. The majority of our patients (10 out of 12 eyes) met and maintained the criteria of success defined in this study through the follow-up without the use of glaucoma medications, and none required additional glaucoma surgery. After MCT, an average IOP in the mid-teens was recorded at six months and one, two, three, four, and five years, and at last follow-up visit, corresponding to an IOP lowering of >30% from baseline. Previous studies comparing the results of MCT to conventional angle surgery (goniotomy and segmental trabeculotomy) reported high rates of medication-free postoperative success after MCT in the short term [14,15,16,17] and in the long-term [11,18], suggesting that a single 360° trabeculotomy may be at least equally or more effective than repeated conventional angle surgery procedures [14,15,16,17,19]. Multiple surgical procedures increase the risk of surgery-related complications (i.e., iris damage and cataract) and expose the children to the consequences of repeated general anesthesia [20]. A recent metanalysis by Ling et al. confirmed that, compared with conventional trabeculotomy, MCT resulted in better IOP control, higher success rate, and fewer medications in the early postoperative follow-up period (3 to 12 months) [21]. In a large number of pediatric glaucoma cases, encompassing various diagnoses, Berger et al. reported a higher IOP reduction at one year after MCT compared to conventional angle surgery by goniotomy and trabeculotomy [16]. In this study, the percentage of IOP reduction at one year was 52%, similar to one reported in our study (58%) at the same time point, and in other studies reporting the short term IOP reduction (6 to 24 months) after MCT: El Sayed and Gawdat (54%) [14], Sarkisian et al. (57%) [12], Temkar et al. (49%) [17], and Girkin et al. (52%) [13].

Several factors may explain the high percentage of SC cannulation and IOP lowering success rate reported in our study. First, our analysis was focused on a homogeneous population of children with isolated trabeculodysgenesis. Second, most of the patients included presented unilateral PCG with age at surgery of more than six months, possibly suggesting the presence of less abnormal anatomy of the SC than the one found in children presenting glaucoma at birth. Finally, all surgical procedures were conducted by a single surgeon experienced with ab-externo canaloplasty.

Trabeculotomy for PCG has witnessed a progressive evolution from segmental to circumferential incision, from the employment of metal instruments to modified monofilament sutures, and finally, dedicated microcatheter. During the last decade, MCT has been proposed as the procedure of choice for childhood glaucoma as it is associated with a higher success rate, lower IOP, reduced medication use, low risk of complications, and more favorable visual outcome compared to traditional angle surgery by goniotomy or segmental trabeculotomy [16,18]. Following MCT, the reported IOP lowering success rates varies from 49 to 92%, according to the different criteria adopted to define success in the various studies [14,15,16,17,19], whereas, after segmental trabeculotomy, the probability of success after a single operation varies from 31 to 50% [11,14,16,18,19]. This difference may have a twofold explanation: with traditional trabeculotomy, using rigid Harms probe, approximately 90°–120° of the SC may be opened with a single operation, compared to the 360° canal opening that may be achieved using a suture or a catheter; secondly using a suture, and moreover, with an illuminated catheter, the surgeon may be sure to cannulate the entirety of the SC or at least an extensive length of its course, being certain of the correct anatomical position of the device, therefore reducing the risk of complications related to SC miscanulation or false passage (i.e., iris damage, cyclodialysis, lens damage, sub-retinal misdirection) [9,22]. In our series, complete cannulation of SC was achieved in all treated eyes with only a few complications observed: transient hyphema and anterior chamber shallowing. Similarly, sporadic cases of severe complications are reported in the literature after MCT (i.e., cataract, endophthalmitis) [14]. It is noteworthy that none of the patients in our study developed lens opacities after surgery.

There is evidence of greater IOP lowering after complete circumferential trabeculotomy than partial (180° or more) trabeculotomy [12,15,23]. Using an illuminated microcatheter, the percentage of complete trabeculotomy varies according to the surgeon’s experience and the treated cases’ selection. In the early description of the MCT technique, Sarkisian achieved complete 360° trabeculotomy in 75% of the PCG patients treated [12], similar percentages with the same technique were reported by Temkar et al. (80%) [17], Berger at al. (80%) [16], Shakrawal et al. (80%) [15], and less frequently by Girkin et al. (25%) [19] and Rojas et al. (21%) [23]. Likewise, a high percentage of circumferential trabeculotomy may be achieved using a monofilament suture [11]. The relevance of achieving an extensive trabecular and SC opening at first surgery may recommend using an illuminated-microcatheter rather than a monofilament suture to facilitate and improve the accuracy of this procedure. However, no comparative analysis of the rate of SC cannulation using both devices is currently available. Furthermore, using the temporal approach adopted in this study, the superior bulbar conjunctiva and sclera are spared for future filtering surgery should it be required for IOP control.

Surgical advances in the treatment of pediatric glaucoma reflect the improvement in the visual outcomes reported in several studies investigating the visual prognosis in these children. A useful visual acuity (>20/200) may be achieved in the majority of the eyes affected by various pediatric glaucoma subtypes [24]. Children with PCG are reported to have a better visual prognosis than those affected by secondary forms of glaucoma, with 70% of the eyes achieving a VA > 20/70 [24]. Similarly, in our study, eyes with PCG showed a mean VA of 0.25 logMAR (20/32) at the last follow-up visit with low refractive error and surgery-induced astigmatism. Due to the frequency of unilateral cases in our study, management of amblyopia was required in the majority of patients.

Interestingly, following the IOP reduction achieved after surgery, we observed an equal increment of the AXL with time in the operated eyes and fellow eyes of children with unilateral PCG. Furthermore, during the length of time encompassed by our study, none of the patients with unilateral PCG developed elevated IOP in the non-affected eye.

The strength of our study is the homogeneity of the childhood glaucoma cases included in the analysis and the longer follow-up time than many of the published reports. Limitations are the retrospective nature, common to most studies, small number of patients and lack of a control population.

In conclusion, our study shows that MCT is highly effective and safe in PCG, providing IOP control and stability at more than two years after surgery without the need for further glaucoma surgery, with absence of significant complications, and good visual outcomes.

## Figures and Tables

**Table 1:** Characteristics of the study population.

Patient	Sex	Ethnicity	Eye	Age at Surgery (Months)	Pre-Op. IOP (mmHG)	Pre-Op. AXL (mm)	Haab Striae	Degrees of Trab.	Intraop. Events	IOP (Months of F.U.) (mmHg)	Glaucoma Drugs
1	F	Caucasian	R	7	30	22.45	Yes	360°	-	14 (104)	No
2	M	Hispanic	R	4	35	23.00	Yes	360°	2nd scleral incision	14 (55)	No
			L	5	30	22.00	No	360°	-	14 (55)	No
3	F	Caucasian	L	6	28	22.14	Yes	360°	Viscoelastic inj.	17 (52)	No
4	F	Caucasian	L	17	58	23.90	No	360°	-	18 (46)	Brinzolamide and travoprost
5	M	Pakistani	L	3	30	22.38	Yes	360°	2nd scleral incision	11 (24)	No
6	F	Caucasian	R	7	30	22.05	Yes	360°	-	14 (100)	No
7	F	Caucasian	L	6	28	22.10	Yes	360°	-	14 (47)	No
8	M	Caucasian	R	3	35	22.90	Yes	360°	-	16 (121)	No
			L	3	28	23.22	Yes	360°	2nd scleral incision	16 (121)	No
9	F	Caucasian	L	11	50	23.75	No	360°	Viscoelastic inj.	17 (49)	Tafluprost
10	M	Indian	R	3	30	22.18	Yes	360°	-	11 (24)	No

Pre-op = Pre-operative; Trab = trabeculotomy extension; Intraop = intraoperative; Inj = injection; IOP = intraocular pressure; F.U. = follow up.

## Data Availability

Data may be made available upon request.
